# Antioxidant and Anti-Inflammatory Activities of *Agrimonia pilosa* Ledeb. Extract

**DOI:** 10.1155/2020/8571207

**Published:** 2020-06-15

**Authors:** Choon Young Kim, Qi-Ming Yu, Hyun-Joo Kong, Joo-Yeon Lee, Kyung-Mi Yang, Jung-Sook Seo

**Affiliations:** ^1^Department of Food and Nutrition, Yeungnam University, Gyeongsan, Gyeongbuk 38541, Republic of Korea; ^2^Department of Nutrition and Food Hygiene, School of Public Health, Guilin Medical University, Guilin, Guangxi 541004, China; ^3^Faculty of Herbal Food Cooking & Nutrition, Daegu Haany University, Gyeongsan, Gyeongbuk 38610, Republic of Korea

## Abstract

The purpose of this study is to investigate the effect of *Agrimonia pilosa* Ledeb. extract (APLE) on lipopolysaccharide- (LPS-) induced cell damage in hepatocytes with a focus on antioxidant and anti-inflammatory activities. Total antioxidant and anti-inflammatory activities of APLE itself were analyzed and phytochemical analysis was performed. Moreover, inhibitory effects of APLE on LPS-induced oxidative stress and inflammation were assessed in human HepG2 hepatocytes. APLE was found to exert *α*,*α*-diphenyl-*β*-picrylhydrazyl (DPPH), 2,2′-azino-bis(3-ethylbenzothiazoline-6-sulphonic acid) (ABTS), and nitrite scavenging activities and reducing power in a dose-dependent manner. The total phenolic and flavonoid contents of APLE were 44.30 ± 1.61 mg GAE/g and 29.65 ± 1.81 mg QE/g, respectively. HPLC analysis revealed that gallic acid is the major phenolic compound in APLE, followed by rutin, genistein, taxifolin, quercetin, luteolin, and apigenin, in descending order. Treatment of 100 and 200 *μ*g/mL APLE significantly reduced LPS-stimulated intracellular reactive oxygen species production to the basal level without any cytotoxicity. Oppositely, APLE reversed LPS-suppressed expression of glutathione peroxidase gene and protein. Consistent with this result, APLE suppressed LPS-triggered expression of proinflammatory cytokine genes in a dose-dependent manner. These results reinforce the fact that the antioxidant and anti-inflammatory activity of APLE helps protect hepatocytes from LPS. Thus, APLE may be utilized as a bioactive ingredient in functional foods.

## 1. Introduction

Lipopolysaccharide (LPS), a glycolipid found in the outer membrane of the Gram-negative bacterial cell wall, is an endotoxin that causes hepatic damage and liver failure [1, 2]. Bacterial LPS is absorbed by the intestine, enters systemic circulation, and is taken up by hepatocytes for detoxification [1, 3]. Liver injury caused by LPS is due to increased oxidative stress and a chronic inflammatory response by proinflammatory cytokines such as tumor necrosis factor-*α* (TNF-*α*), interleukin-6 (IL-6), and IL-1*β* [4, 5].

Oxidative stress in the pathogenesis of LPS-induced hepatic damage is associated with increased generation of ROS and/or decreased levels of endogenous antioxidant enzymes including glutathione peroxidase (GpX), catalase, and superoxide dismutase (SOD) [6]. Oxidative stress exacerbates the inflammatory responses and vice versa [7]. Increased production of ROS enhances the inflammatory response by upregulating the genes related to immune and inflammatory cytokines through activation of the nuclear factor-*κ*B, a redox-sensitive nuclear transcription factor [8]. Therefore, an effective way to prevent LPS-induced hepatic damage is the administration of antioxidants [9].

Accumulating evidence supports the hypothesis that individual antioxidants effectively protect the liver from LPS-induced hepatic damage. Vitamin C supplement significantly lowered LPS-induced oxidative damage in the liver of Guinea pigs [10]. Melatonin feeding in rat [11] and selenium treatment [4] also exhibited a hepatoprotective activity by suppressing the production of ROS and nitric oxide (NO) stimulated by LPS. *N*-acetyl-l-cysteine, a precursor of glutathione, also inhibited oxidative stress stimulated by LPS [12, 13]. Taurine administration in rats has been demonstrated to protect LPS-stimulated liver injury through antioxidant and anti-inflammatory activity [14].

Even though a single antioxidant is able to protect the liver from hepatotoxicity caused by LPS, there is an increasing interest in finding natural bioactive compounds with an ability to reduce liver damage due to their superior efficacy. Most natural antioxidants isolated from vegetables, fruits, tea, spices, and medical herbs have been demonstrated to show synergistic or additive activity because they contain multiple bioactive components [15]. Indeed, the mixture of bioactive compounds in plant extracts synergistically augments their antioxidant activity [16]. Thus, there are many efforts to investigate the protective effects of natural products against LPS-stimulated oxidative stress and inflammation. Morita et al. reported that nutmeg showed the strongest hepatoprotective potential among 21 different spices tested and they established that nutmeg extract contains multiple bioactive components, including myristicin [17]. Administration of argan oil to mice injected with LPS attenuated the oxidative stress and inflammation in the liver, as demonstrated by the improved activities of antioxidant enzymes such as Gpx, catalase, and SOD and reduced the expression of proinflammatory cytokines such as TNF-*α* and IL-6 [18].

*Agrimonia pilosa* Ledeb. (APL), an herbaceous perennial plant, is widely used in traditional medicine because of its multiple biological functions including anticancer, antioxidant, anti-inflammatory, and antidiabetic properties. Anticancer activity of APL has been supported by the finding that agrimoniin, a tannin found in APL, inhibited tumor growth in mice [19]. Moreover, it is believed that APL helps reduce feelings of tiredness and fatigue [20] so APL is used to treat asthenia and inflammations in allergic disease [21]. The anti-inflammatory activity of APL in macrophages has been reported due to its NO scavenging ability [22]. The flavonoid and triterpenoid compounds from *Agrimonia pilosa* Ledeb. were also reported to have showed beneficial effects on type-2 diabetes mellitus by inhibiting oxidative stress and hyperglycemia [23]. Even though multiple biological functions of APL have been previously reported, the effect of APL on LPS-induced cellular damage in hepatocytes had not yet been investigated. Thus, this study focused on determining the antioxidant and anti-inflammatory activities of APL and its bioactive compounds as well as the *in vitro* effect of APLE on oxidative stress and inflammation, the known pathogenic causes of hepatotoxicity.

## 2. Materials and Methods

### 2.1. Materials

Dulbecco's Modified Eagle's Medium (DMEM), 0.25% trypsin-EDTA, penicillin-streptomycin, sodium pyruvate, and fetal bovine serum (FBS) were purchased from Thermo Scientific (Waltham, MA, USA). Antibodies were obtained from Santa Cruz Biotechnology (Santa Cruz, CA, USA). Lipopolysaccharide (LPS), dimethyl sulfoxide (DMSO), and other reagents were purchased from Sigma-Aldrich Co. (St. Louis, MO, USA).

### 2.2. Preparation of *Agrimonia pilosa* Ledeb. Extract (APLE)

Leaves of *Agrimonia pilosa* Ledeb. were purchased from a local food company in Gyeongsang in Korea. Extraction was carried out thrice by mixing *Agrimonia pilosa* Ledeb. (50 g) with 500 mL of 60% (v/v) ethanol at 70°C for 3 h, and the extract was filtered. Supernatant was evaporated at 80°C using a rotary evaporation concentrator (Rotavapor R-210, Buchi, Switzerland) to remove the extract solvent. The powdered form of *Agrimonia pilosa* Ledeb. extract (APLE) was obtained after freeze-drying (FD8512 freeze drier, ilShinbiobase Co. Ltd, Gyounggi-do, Korea). APLE was stored at −20°C for further analysis.

### 2.3. Determination of Total Antioxidant Capacity of APLE

#### 2.3.1. DPPH Radical Scavenging Activity Assay

Electron donating ability was measured using 1,1-diphenyl-2-picrylhydrazyl (DPPH) according to the method of Blois et al. [24]. Briefly, 1 mL of 0.2 mM DPPH solution was added to 2 mL of APLE prepared for each concentration. After mixing it using a mixer for 10 sec, the mixture was incubated at 37°C for 30 min. The absorbance of the reaction solution was measured using an absorption spectrophotometer (Hitachi UV-2001, Tokyo, Japan) at 517 nm. The electron-donating ability expressed the difference in absorbance before and after sample addition in %.

(1) $$\begin{array}{c} \text{DPPH radical scavenging activity }\left(\text{\%}\right)\;=\left[1-\frac{\left({\text{Absorbance}}_{\text{sample}}\;-\;{\text{Absorbance}}_{\text{blank}}\right)}{{\text{Absorbance}}_{\text{control}}}\;\right]\times 100. \end{array}$$

#### 2.3.2. ABTS Radical Scavenging Activity Assay

The ABTS scavenging activity was determined by the method of Re et al. [25]. The ABTS solution was produced by mixing 7.4 mM 2,2′-azino-bis (3-ethylbenzothiazoline-6-sulfonic acid) diammonium salt (ABTS) with 2.6 mM potassium persulfate and then diluted with phosphate buffer saline (pH 7.4) until the absorbance of the ABTS solution was 0.70 ± 0.03 at 732 nm. After reacting 950 *μ*L of diluted ABTS solution with 50 *μ*L of sample for 10 min in the dark, the absorbance was measured at 732 nm using a spectrophotometer (Hitachi). The ABTS radical scavenging activity was calculated as the ratio of the decrease in absorbance between the sample and no sample.

#### 2.3.3. Superoxide Dismutase- (SOD-) Like Activity Assay

SOD-like activity was measured by measuring the amount of pyrogallol that catalyzes the conversion to hydrogen peroxide according to the method of Marklund et al. [26]. The reaction mixture was prepared by mixing 0.2 mL of sample and 3 mL of 50 mM Tris-HCl buffer (pH 8.5) with 10 mM EDTA and 0.2 mL of 7.2 mM pyrogallol. The reaction mixture was incubated at 25°C for 10 min, and then the reaction was stopped by 0.1 mL of 1 N HCl. Absorbance of the reaction mixture was determined at 420 nm. SOD-like activity was calculated as the ratio of the decrease in absorbance between the sample and control.

#### 2.3.4. Reducing Power Assay

The reducing power was measured by a modification of the method of Wong et al. [27]. To each 0.5 mL of sample solution, 1 mL of 0.2 M phosphate buffer (pH 6.6) and 1 mL of 1% potassium ferricyanide were added to make a reaction mixture. The reaction was allowed to proceed at 50°C for 30 min and then the mixture was cooled to room temperature, followed by adding 1 mL of 10% TCA solution. After incubating for 10 min, 0.5 mL of the reaction mixture was mixed with 1 mL of distilled water and 0.5 mL of 0.1% FeCl_3_ and then the absorbance was measured at 700 nm.

### 2.4. Nitrite Scavenging Ability Assay

The nitrite scavenging activity of each extract was measured according to the method of Kato et al. [28]. Specifically, the procedure was as follows: 1 mL of each concentration of samples was added to 2 mL of 1 mM NaNO_2_ solution, and the pH of the reaction mixture was adjusted to 1.2 and 3.0 using 0.1 N HCl (pH 1.2) and 0.1 M citric acid buffer solution, respectively. This was followed by adjusting the final volume of the reaction mixture to 10 mL. Then, 1 mL of the reaction mixture obtained by reacting at 37°C for 1 h was taken, and 5 mL of 2% acetic acid was added, followed by mixing with 0.4 mL of Griess reagent. After incubating at room temperature for 15 min, the absorbance was measured at 520 nm using an absorption spectrophotometer. The nitrite scavenging activity was (%) = [1 − (S − B)/C] × 100, where C is the absorbance of the control, S is the absorbance of the sample without the Griess reagent, and B is the absorbance of the sample with the Griess reagent.

### 2.5. Determination of Total Antioxidant Compounds

#### 2.5.1. Total Phenolic Content Assay

Total phenolic content was determined using the Folin–Ciocalteu's reagent method [29]. Gallic acid was used as a standard, and phenolic content was expressed as mg gallic acid equivalents/g (mg GAE/g).

#### 2.5.2. Total Flavonoid Content Assay

Total flavonoid content was determined by colorimetry using the method of Moreno et al. [30]. The reaction mixture was prepared by adding 0.1 mL of APLE to 4.3 mL of 80% ethanol solution containing 10% aluminum nitrate and 1 M potassium acetate. This reaction mixture was incubated at 25°C for 40 min, and then the absorbance was measured at 415 nm using a spectrophotometer (Hitachi). The total flavonoid content of APLE was determined from a calibration curve obtained using quercetin as a standard.

### 2.6. HPLC Analytical Method of Phenolic Compounds in *Agrimonia pilosa* Ledeb. Extract

Phenolic compounds were analyzed by HPLC on a Shimadzu LC-20A with UV detector (SPD-20A, Shimadzu, Kyoto, Japan) and equipped with an InertSustain C18 column (4.6 × 250 mm; 5 *μ*m) (GL Sciences, Tokyo, Japan). APLE was redissolved in methanol. All samples and standard compounds were filtrated through a 0.22 *μ*m syringe filter membrane and directly injected into the system for the analysis. The gradient elution mobile phase consisted of solvent A (0.1% acetic acid in water) and solvent B (methanol). The total flow rate was 1 mL/min and the sample injection volume was 20 *μ*L. Gradient elution conditions were as follows: 0–5 min, 5 to 10% B; 5–10 min, 5 to 25% B; 10–15 min, 25 to 30% B; 15–20 min, 30 to 35% B; 20–40 min, 35 to 40% B; 40–65 min, 60% B; 65–110 min, 100% B; 110–115 min, 0% B.

### 2.7. Cell Culture and Treatment Conditions

HepG2 cell, a human hepatoma cell line, was obtained from American Type Culture Collection (Manassas, VA). HepG2 cells were cultured in 10% FBS-DMEM medium supplemented with 100 units/mL penicillin and 100 mg/mL streptomycin at 37°C under 5% CO_2_. When cell confluency reached about 80%, cells were treated with APLE in the absence and presence of 100 ng/mL LPS for 24 h.

### 2.8. Detection of Intracellular Oxidative Stress

A 2′,7′-dichlorofluorescein diacetate (DCFDA) assay was performed to estimate intracellular reactive oxygen species. After 24 h of HepG2 cells treatment with 0, 100, 200, and 400 *μ*g/mL of APLE together with 0 and 100 ng/mL LPS, cells were incubated with phosphate-buffered saline (PBS) containing 120 *μ*M DCFDA for 60 min at 37°C. The absorbance was determined at 488 nm excitation and 525 nm emission using a fluorescence plate reader (VICTOR X3, PerkinElmer, Turku, Singapore).

### 2.9. Measurement of Cell Viability by MTT Assay

Cell viability of HepG2 after treatment with APLE and with and without 100 ng/mL LPS was determined by 3-(4,5-dimethyl-thiazol-yl-2)-2,5-diphenyl tetrazolium bromide (MTT) assay. At the time of 60% confluence, HepG2 cells were treated with 0, 100, 200, and 400 *μ*g/mL APLE together with 0 or 100 ng/mL LPS for 24 h. After aspirating the cell culture medium, cells were cultured in 10% FBS–DMEM with 5 mg/mL MTT solution. After 1 h of incubation, formazan, a purple product converted from a tetrazolium salts by the viable cells, was spectrophotometrically monitored at 595 nm. Cell viability was calculated based on the following equation:

(2) $$\begin{array}{c} \text{cell viability }\left(\text{\% of control}\right)\;=\;\frac{\left({\text{absorbance}}_{\text{sample}}\;-{\text{ absorbance}}_{\text{blank}}\right)}{\left({\text{absorbance}}_{\text{control}}\;-\;{\text{absorbance}}_{\text{blank}}\right)}\;\times 100. \end{array}$$

### 2.10. Estimation of Cell Toxicity by Lactate Dehydrogenase (LDH) Activity Assay

The cytotoxicity of HepG2 treated with APLE and LPS was determined with an LDH activity assay using a commercial test kit (Pierce LDH cytotoxicity assay kit, #88953, Thermo Fisher Scientific, Waltham, MA, USA). LDH activity in the cell culture medium was estimated according to the manufacturer's protocol. HepG2 cells were treated with different concentrations of APLE and LPS (0 and 100 ng/mL) for 24 h and then medium was collected for analysis. Collected medium was added to reconstituted substrate mix at room temperature for 30 min and then the reaction was stopped with a stop solution. The activity of LDH was spectrophotometrically obtained at a wavelength of 490 nm using a microplate reader (Epoch, BioTek, Winooski, VT, USA).

### 2.11. Assessment of Gene Expression Related to Inflammation and Oxidative Stress by Quantitative RT-PCR

Total RNA was extracted from HepG2 cells using TriZol reagent (Invitrogen, Carlsbad, CA, USA) and cDNA was synthesized using the SuperScript II kit (Invitrogen). Using this cDNA as a template, the gene of interest was amplified by quantitative RT-PCR using Power SYBR Green PCR master mix and thermocycler (Applied Biosystems, Foster City, CA, USA). The nucleotide sequence of the primers for human genes used in the PCR assay was as follows: tumor necrosis factor-*α* (forward, 5′-CCC AGG CAG TCA GAT CAT CTT C-3′; reverse, 5′-AGC TGC CCC TCA GCT TGA-3′), interleukin-6 (forward, 5′-GGT ACA TCC TCG ACG GCA TCT-3′; reverse, 5′-GTG CCT CTT TGC TGC TTT CAC-3′), interleukin-1*β* (forward, 5′-TGG CAA TGA GGA TGA CTT GTT C-3′; reverse, 5′-CTG TAG TGG TGG TCG GAG ATT-3′), glutathione peroxidase 1 (forward, 5′-GGT TTT CAT CTA TGA GGG TGT TTC C-3′; reverse, 5′-GCC TTG GTC TGG CAG AGA CT-3′), and *β*-actin (forward, 5′-TGA CGG GGT CAC CCA CAC TGT GCC CAT CTA-3′; reverse, 5′-CTA GAA GCA TTT GCG GTG GAC GAT GGA GGG-3′).

### 2.12. Western Blot Analysis

The cells were incubated with APLE in the absence and presence of 100 ng/mL LPS for 24 h. The cells were rinsed with ice-cold PBS, and harvested by centrifugation as previously described [31]. The cells were lysed with the lysis buffer composed of 100 mM Tris-HCl buffer (pH 7.5–8.0) containing 100 mM NaCl, 0.5% TritonX-100, protease inhibitor cocktail (Sigma-Aldrich Co.), 1 mM sodium orthovanadate, and 10 mM sodium fluoride. The cell lysates were centrifuged to yield a clear lysate. Supernatant was collected, and protein concentration was estimated by the Bradford method (Bio-Rad Laboratories, Hercules, CA, USA). The proteins were separated by 10% SDS-PAGE and were transferred to a polyvinylidene difluoride (PVDF) membrane. The membrane was incubated for 1 h in blocking solution (5% nonfat dried milk in Tris-buffered saline with 0.1% Tween 20 buffer). Immunoblot assay was performed by anti-TNF-*α* (anti-TNF-*α*; Santa Cruz Biotechnology), anti-GpX (anti- GpX; Santa Cruz Biotechnology), and anti-*β*-actin (Santa Cruz Biotechnology) antibodies at 4°C overnight. After being washed in Tris-buffered saline with 0.1% Tween buffer, the membrane was incubated in appropriate secondary antibodies. The membrane was developed using ECL Prime Western Blotting Detection Reagent (GE Healthcare, Milwaukee, WI, USA) on X-ray film. Experiments were repeated in triplicate. ImageJ software was used to determine the band densities of proteins relative to *β*-actin.

### 2.13. Statistical Analysis

All experimental results are expressed as the mean ± standard deviation of three independent experiments in triplicate. Statistical analysis was performed by Duncan's multiple range test after analysis of variance using Statistical Analysis System (SAS 9.4) software (SAS Institute, Cary, NC, USA). Statistical significance was tested at a level of *α* = 0.05. Correlations among antioxidant and anti-inflammatory activities and *in vitro* gene expressions were obtained by calculating Pearson's correlation coefficient.

## 3. Results

### 3.1. Antioxidant and Anti-Inflammatory Activities of *Agrimonia pilosa* Ledeb. Extract (APLE)

**Figure 1:**
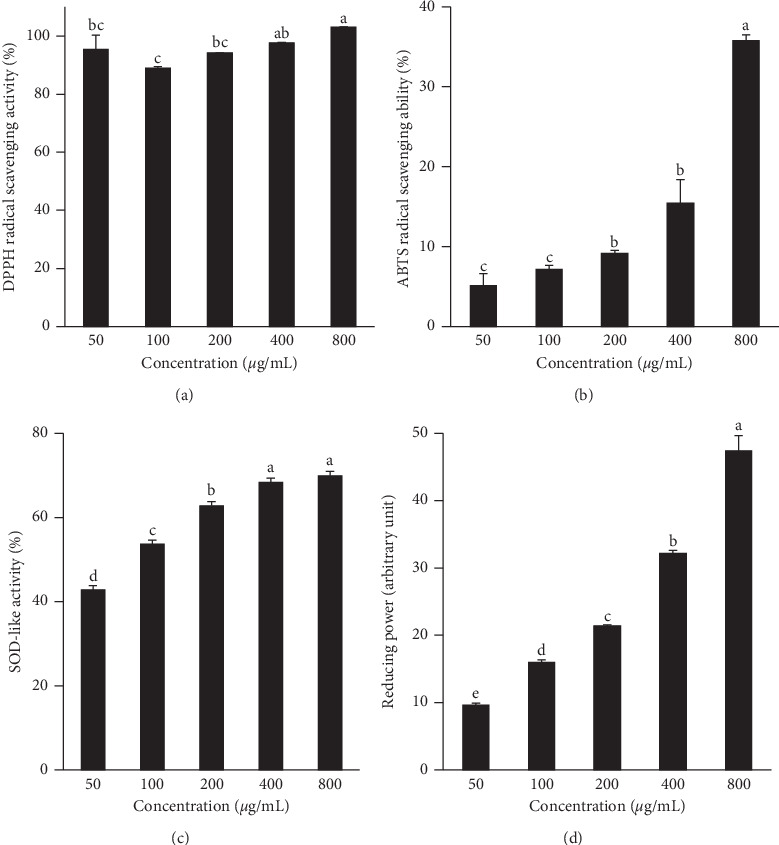
Antioxidant activities of *Agrimonia pilosa* Ledeb. extract (APLE). Free radical scavenging activities of APLE were determined by DPPH radical (a) and ABTS radical (b) scavenging activities. Superoxide dismutase- (SOD-) like activity (c) and reducing power (d) were estimated by the pyrogallol method and ferric-reducing antioxidant power assay, respectively. Values are expressed as means ± standard deviation (SD) from three experiments. Different letters indicate a significant difference among groups (*p* < 0.05).

In order to determine antioxidant activity of APLE, the free radical scavenging activity of APLE ranging from 50 to 800 *μ*g/mL was determined using *α*,*α*-diphenyl-*β*-picrylhydrazyl (DPPH) and 2,2′-azino-bis(3-ethylbenzothiazoline-6-sulphonic acid) (ABTS) radicals. The DPPH and ABTS radical scavenging activities were increased as the concentration of APLE increased. In particular, the DPPH radical scavenging activity of APLE at 800 *μ*g/mL was almost 100% (Figure 1(a)).  Figure 1(b) shows that the ABTS radical scavenging activities of APLE at 400 *μ*g/mL were 15.46 ± 1.69%.

As superoxide dismutase (SOD) is an important antioxidant enzyme catalyzing the dismutation of the superoxide radical into oxygen and hydrogen peroxide, determination of SOD-like activity of compounds is an appropriate method to estimate antioxidant activity. When SOD-like activity of APLE at different concentrations was measured, dose-dependent responses were observed up to 400 *μ*g/mL (Figure 1(c)). APLE at 400 and 800 *μ*g/mL showed approximately 68% of SOD-like activity.

The total antioxidant activity can be determined by the ferric-reducing antioxidant power assay, which is based on a redox-linked colorimetric reaction, in which ferric iron (Fe^3+^) is reduced to ferrous iron (Fe^2+^). The reducing power of APLE was significantly increased in a dose-dependent manner, in the range of 10 to 50 arbitrary units (Figure 1(d)).

**Figure 2:**
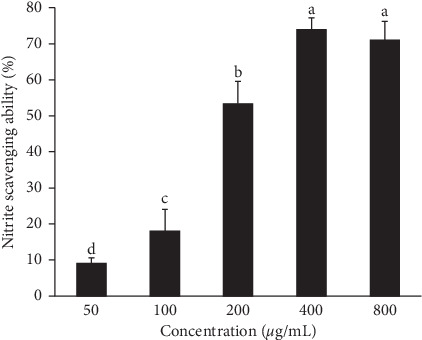
Anti-inflammatory activity of *Agrimonia pilosa* Ledeb. extract (APLE). The anti-inflammatory property of APLE was measured using a nitrite scavenging activity assay. Values are expressed as means ± standard deviation (SD) from three experiments. Different letters indicate a significant difference among groups (*p* < 0.05).

Nitrite (NO_2_^−^) level is known to be a critical marker for inflammation [32]. As shown in Figure 2, APLE exhibited a concentration-dependent trend in its ability to scavenge nitrate. The highest nitrate scavenging activity of 73.9 ± 3.241% was obtained at a concentration of 400 *μ*g/mL.

### 3.2. Total Phenolic and Flavonoid Contents in APLE

**Figure 3:**
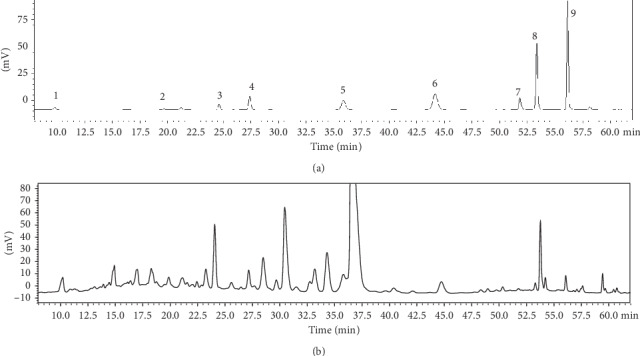
HPLC chromatogram of phenolic contents in extract of *Agrimonia pilosa* Ledeb. (a) Chromatogram of standard references and (b) sample. Peaks (1) gallic acid, (2) epigallocatechin gallate, (3) daidzin, (4) taxifolin, (5) rutin, (6) quercitrin, (7) luteolin, (8) quercetin, and (9) apigenin.

In order to identify the compounds responsible for the antioxidant and anti-inflammatory properties of APLE, the content of plant phenolic compounds and flavonoid and individual bioactive compounds were measured. The total phenolic and flavonoid contents in APLE were 44.30 ± 1.61 mg GAE/g and 29.65 ± 1.81 mg QE/g, respectively. HPLC analysis exhibited the phenolic profile of APLE and identified seven compounds (Figure 3). The concentrations of seven phenolic compounds in APLE were the following, in descending order: gallic acid, rutin, genistein, taxifolin, quercetin, luteolin, and apigenin (Table 1). The major compound in APLE was gallic acid and its concentration was estimated at 11.27 ± 0.63 mg/g. The concentrations of rutin, genistein, taxifolin, quercetin, luteolin, and apigenin were 3.12, 2.45, 1.89, 0.39, 0.15, and 0.14 mg/g, respectively.

### 3.3. Inhibitory Effect of APLE on Lipopolysaccharide- (LPS-) Induced Oxidative Stress Without Cytotoxicity

**Figure 4:**
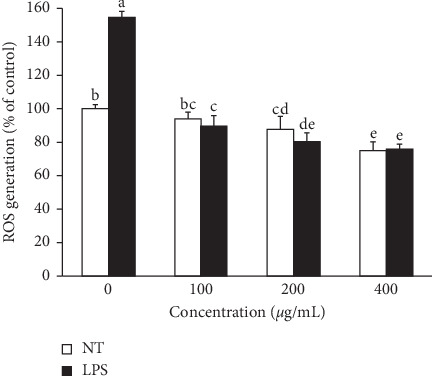
Effect of *Agrimonia pilosa* Ledeb. extract (APLE) and lipopolysaccharide (LPS) on the production of intracellular reactive oxygen species (ROS) in HepG2 cells. To assess the level of intracellular ROS, a dichlorofluorescein diacetate (DCFDA) assay was performed using HepG2 cells treated with APLE at concentrations ranging from 0 to 400 *μ*g/mL in the presence and absence of 100 ng/mL of LPS for 24 h. Means that do not share any letters are significantly different at *p* < 0.05. (NT: nontreated cells).

The effect of APLE on oxidative stress was determined by dichlorofluorescein diacetate (DCFDA) assay (Figure 4). The assay is based on the oxidation of the nonfluorescent DCFH upon reaction with reactive oxygen species (ROS) to form the fluorescent DCF in HepG2 cell treated with LPS. The cells stimulated with LPS showed significantly higher intracellular reactive oxygen species (ROS) production by 1.6 folds compared to the nontreated cells (NT). However, APLE treatment blunted LPS-stimulated ROS generation in a dose-dependent manner. ROS production in cells treated with APLE at 100 and 200 *μ*g/mL without LPS was similar to that in NT. Interestingly, APLE treatment in the presence of LPS showed a slightly lower ROS level compared with NT. Cells treated with APLE at a concentration of 400 *μ*g/mL had the lowest ROS levels regardless of LPS treatment. In the presence of LPS, APLE treatment at 200 or 400 *μ*g/mL showed statistically similar suppression of LPS-induced ROS generation.

In order to determine whether the reduction of intracellular ROS level by APLE treatment shown in Figure 4 was due to APLE cytotoxicity, cell viability and cytotoxicity were estimated in HepG2 cells treated with APLE in the absence or presence of LPS (data not shown). Without APLE treatment, LPS slightly, but not significantly, reduced cell viability. However, APLE at 100 and 200 *μ*g/mL did not affect cell viability without LPS but protected cells from LPS-lowered viability. Up to 200 *μ*g/mL of APLE and LPS alone or in combination did not cause any cytotoxicity. Thus, the cell viability and cytotoxicity assays suggested that, regardless of the presence of LPS, concentrations of 100 and 200 *μ*g/mL are safe levels for APLE treatment of HepG2 cells.

### 3.4. Inhibitory Effect of APLE on LPS-Altered Expressions of Genes and Proteins Related to Proinflammatory Cytokines and Antioxidant Enzyme

**Figure 5:**
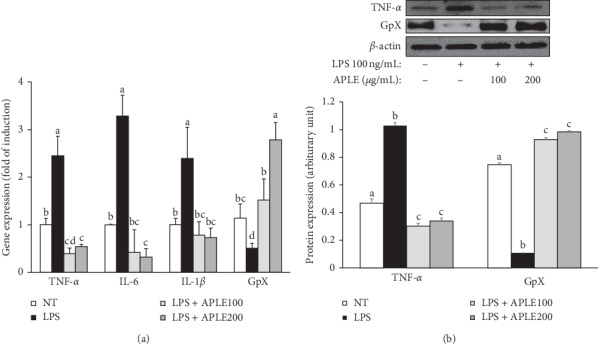
Effect of *Agrimonia pilosa* Ledeb. extract (APLE) on the levels of genes and proteins related to inflammation in lipopolysaccharide- (LPS-) induced HepG2 cells. HepG2 cells were treated with APLE (0, 100, and 200 *μ*g/mL) in the presence and absence of LPS (100 ng/mL) for 24 h. (a) Quantitative RT-PCR for the estimation of gene expression related to inflammation (TNF-*α*, IL-6, and IL-1*β*) and an antioxidant enzyme, glutathione peroxidase (GpX). (b) Protein levels of TNF-*α* and GpX were assessed by immunoblot assay. The level of *β*-actin was shown as a loading control and used for normalization of the band intensity.

To examine the effect of APLE on LPS-induced dysregulation of gene and protein expression, quantitative RT-PCR and immunoblot analyses were carried out, respectively. As shown in Figure 5(a), the expression of proinflammatory cytokine genes, tumor necrosis factor-alpha (TNF-*α*), interleukin-6 (IL-6), and IL-1*β* was significantly increased by more than twofold after LPS treatment, compared with corresponding the vehicle control, nontreated cells (NT). However, APLE treatment significantly lowered the gene levels of proinflammatory cytokines. On the other hand, LPS treatment significantly reduced the expression of the antioxidant enzyme glutathione peroxidase (GpX), whereas APLE increased the GpX gene expression reduced by LPS in a dose-dependent manner. Consistent with this result, protein levels of TNF-*α* in APLE treated groups were significantly lower than the LPS-treated group (Figure 5(b)). LPS-suppressed GpX protein level was dose-dependently elevated by APLE treatment.

### 3.5. Correlation between Antioxidant and Anti-Inflammatory Activities and *In Vitro* Gene Expression of APLE

In order to explore the possible relationships among antioxidant and anti-inflammatory activities and *in vitro* gene expression, a Pearson's correlation coefficients was obtained (Table 2). Antioxidant activity assays, SOD and FRAP, were highly correlated with anti-inflammatory NO activity (*p* < 0.001). The expressions of proinflammatory genes, TNF-*α*, IL-6 and IL-1*β,* were strongly correlated with each other, in which Pearson's correlation coefficients *r* between TNF-*α* expression with both IL-6 and IL-1*β* expression were 0.959 and 0.886, respectively. A positive correlation between antioxidant and anti-inflammatory activities and GpX gene expression was observed, in contrast to a negative relationship between antioxidant and anti-inflammatory activities and proinflammatory gene expression.

## 4. Discussion

Compounds with antioxidant and anti-inflammatory activities may alleviate LPS-induced deterioration in hepatocytes. In hepatocytes exposed to lipopolysaccharide (LPS), the inflammatory responses are accompanied by elevated oxidative stress, leading to hepatic dysfunction and damage. *Agrimonia pilosa* Ledeb. extract (APLE) is known to possess a beneficial effect on human health [19–23, 33, 34]. Thus, the present study was performed to examine the effect of APLE on LPS-induced dysfunction in hepatocytes, focusing especially on oxidative stress and inflammatory responses.

Figure 4 shows that LPS causes a significant level of oxidative stress but APLE treatment significantly lowers LPS-induced intracellular reactive oxygen species (ROS) levels. It is observed that suppression of intracellular ROS levels by APLE was not relevant to the cytotoxicity of APLE. Consistent with ROS levels, the expression of gene and protein encoding glutathione peroxidase (GpX) was significantly downregulated by LPS treatment, whereas APLE eliminated this effect and even elevated LPS-reduced GpX expression (Figure 5). In contrast, genes related to proinflammatory cytokines, such as tumor necrosis factor-*α* (TNF-*α*), interleukin-6 (IL-6), and IL-1*β*, were upregulated by LPS but APLE reversed LPS's deteriorating action.

The results of analyzing the total antioxidant and nitrite scavenging activity of APLE itself (Figures 1 and 2) verified the inhibitory effect of APLE on LPS-stimulated oxidative stress and inflammation in the cell culture system. In agreement with our results, the induction of inflammation and oxidative stress in HepG2 cells treated with LPS has been reported. Kim et al. [4] showed that APLE blocked the LPS-upregulated gene expression of IL-6 and IL-1*β*, but not that of TNF-*α* [33]. The gene expression levels of antioxidant enzymes such as glutathione, GpX, and superoxide dismutase were reduced, whereas proinflammatory cytokine IL-8 secretion and gene expression were significantly increased [35]. Meanwhile, treatment with *N*-acetyl-l-cysteine, a powerful antioxidant, inhibited LPS-induced IL-8 expression and secretion [35].

Polyphenolic compounds and flavonoids are bioactive constituents of natural products with powerful antioxidant capacity, thus exerting protective functions against oxidative damage and providing several health benefits [36]. In accordance with results of the antioxidant activity assay, APLE contains polyphenolic compounds and flavonoids as demonstrated by the colorimetric assay. In addition, HPLC analysis revealed that APLE contains various phenolic compounds; in descending order, the concentration of phenolic compounds decreased as follows: gallic acid, rutin, genistein, taxifolin, quercetin, luteolin, and apigenin (Table 1). Differing from our data, Zhu et al. previously detected quercitrin, rutin, luteolin-7-*O*-*β*-D-glucopyranoside, taxifoliol, and hyperoside [20]. This discrepancy could be due to the difference in the extraction solvents used (60% ethanol versus water). Even though gallic acid, the major phenolic in APLE, has been reported to have antioxidant and anti-inflammatory functions [37], the function of APLE did not solely come from gallic acid. The combination of different phenolic and nonphenolic compounds in APLE additively and synergistically contributes to its antioxidant and anti-inflammatory activities [15, 16].

## 5. Conclusion

Our findings suggest that APLE has the potential to prevent LPS-induced oxidative stress and inflammation due to antioxidant and anti-inflammatory activities of phenolic compounds in APLE. Therefore, APLE may be a good candidate as a functional ingredient for improving and maintaining liver health. Further *in vivo* studies are required to verify the hepatoprotective effect of APLE and to elucidate the molecular mechanisms underlying this function.

## Figures and Tables

**Table 1:** The concentration of phenolic compounds in *Agrimonia pilosa* Ledeb. extract.

Compounds	Concentration^1^ (mg/g)
Gallic acid	11.27 ± 0.63
Rutin	3.12 ± 0.05
Genistein	2.45 ± 0.00
Taxifolin	1.89 ± 0.02
Quercetin	0.39 ± 0.00
Luteolin	0.15 ± 0.00
Apigenin	0.14 ± 0.00

^1^Values are means ± standard deviation (SD).

**Table 2:** Pearson's correlation coefficients between the antioxidant and anti-inflammatory activities and *in vitro* gene expression *of Agrimonia pilosa* Ledeb. extract.

	DPPH	ABTS	FRAP	SOD	NO	TNF-*α*	IL-6	IL-1*β*	GpX
DPPH	1								
ABTS	0.507	1							
FRAP	0.671^*∗∗*^	0.757^*∗∗*^	1						
SOD	0.429	0.546^*∗*^	0.876^*∗∗∗*^	1					
NO	0.534^*∗*^	0.455	0.842^*∗∗∗*^	0.930^*∗∗∗*^	1				
TNF-*α*	−0.495	−0.342	−0.444	−0.245	−0.406	1			
IL-6	−0.501	−0.373	−0.420	−0.189	−0.343	0.959^*∗∗∗*^	1		
IL-1*β*	−0.473	−0.290	−0.395	−0.142	−0.333	0.886^*∗∗∗*^	0.827^*∗∗∗*^	1	
GpX	0.562^*∗*^	0.530^*∗*^	0.852^*∗∗∗*^	0.714^*∗∗*^	0.728^*∗∗*^	−0.349	−0.279	−0.307	1

DPPH: *α*,*α*-diphenyl-*β*-picrylhydrazyl; ABTS: 2,2′-azino-bis(3-ethylbenzothiazoline-6-sulphonic acid); FRAP; SOD: superoxide dismutase-like activity; NO: nitrite scavenging ability; TNF-*α*: tumor necrosis factor-alpha; IL-6: interleukin-6; IL-1*β*: interleukin-1beta; GpX: glutathione peroxidase. ^*∗*^Correlation is significant at *p* < 0.05. ^*∗∗*^Correlation is significant at *p* < 0.01. ^*∗∗∗*^Correlation is significant at *p* < 0.001.

## Data Availability

Experimental data used to support the findings of this study are available from the corresponding author upon request.
